# Effects of In Vitro Maturation on Histone Acetylation in Metaphase II Oocytes and Early Cleavage Embryos

**DOI:** 10.1155/2010/989278

**Published:** 2010-06-20

**Authors:** Ning Wang, Fang Le, Qi-Tao Zhan, Li Li, Min-Yue Dong, Guo-Lian Ding, Chen-Ming Xu, Shi-Wen Jiang, He-Feng Huang, Fan Jin

**Affiliations:** ^1^Department of Reproductive Endocrinology, Women's Hospital, School of Medicine, Zhejiang University, 310006 Hangzhou, China; ^2^Department of Biomedical Science, Mercer University School of Medicine, Savannah, GA 31499, USA; ^3^Department of Obstetrics and Gynecology, Mayo Clinic, Rochester, MN 55905, USA

## Abstract

In vitro maturation (IVM) of oocyte is an effective procedure for avoiding ovarian hyperstimulation syndrome in patients with polycystic ovaries (PCOS) during in vitro fertilization (IVF). To investigate the influences of IVM on epigenetic reprogramming and to search for the possible reasons for the lower rates of fertilization and cleavage in IVM oocytes, we examined the expression of two enzymes controlling histone acetylation, histone acetyltransferase GCN5 (GCN5) and histone deacetylase 1 (HDAC1), as well as their common target, acetyl-histone H3 (Ac-H3), in mouse metaphase II (MII) oocytes and preimplantation embryos. Results showed that IVM downregulated the protein expression of GCN5 in MII oocytes and two-cell embryos and changed the distribution of GCN5 in two-cell embryos. Expression of HDAC1 mRNA in MII oocytes and two-cell embryos decreased in the IVM group. However, none of these changes persisted after two-cell embryos. Levels of Ac-H3 in both oocytes and embryos remained unchanged after IVM. Our studies indicated that IVM could affect the protein and gene expression related to histone acetylation in oocytes and early cleavage embryos. By function of selection, parts of the changes could be recovered in late embryo development.

## 1. Introduction

In vitro fertilization and embryo transfer (IVF-ET) are an effective treatment for infertility [1, 2]. However, the high costs of gonadotropin administration, the risk of ovarian hyperstimulation syndrome (OHSS), and the possible association between repeated ovarian stimulation and hormone-related cancers are the main drawbacks of IVF-ET. 

In-vitro maturation (IVM) offers an alternative to conventional IVF that minimizes medicine administration and avoids ovarian hyperstimulation. Meanwhile, poor responders to gonadotropin stimulation may also benefit from IVM as they do not need to receive a large dosage of gonadotropins. With the cryopreservation of reproductive cells, IVM can offer to preserve fertility in women who are undergoing cancer treatment [3]. So far more than 1000 children have been born from IVM procedures, particularly in the patients with PCOS [4, 5]. However, IVM remains a challenge in mammalian species, especially for human. Some concern has been voiced regarding the safety of this new method with respect to the health of the children [6]. Questions have arisen on whether human oocytes matured in vitro are intrinsically compromised or whether culture conditions are inadequate to support the full developmental potential of the oocytes [7]. 

Oocyte maturation is one of the most critical periods for normal development and differentiation for an individual [8]; however, little is known about the mechanisms that regulate early folliculogenesis and oocyte maturation in human. The oocyte genome is epigenetically reprogrammed during meiosis, which is followed by fertilization, to allow the remarkable transformation from differentiated oocytes into the totipotent embryos of the next generation [9]. Epigenetic reprogramming is a scheduled genome-wide modification that occurs in the periods of gametogenesis and embryogenesis, which regulates the gene activity without alteration of DNA sequences [10]. Epigenetic reprogramming leads to re-establishment of gene imprinting patterns, silences or activates genes systematically, and represents a stage susceptive to the changes of environment [11]. Oocyte growth and maturation appear to be vulnerable to environmental factors that can induce the epigenetic alteration, deregulation gene expression, and ultimately, embryo defects or loss.

Although the technique of IVM in human has been gradually improved, its successful rate remains low compared with IVF. The changes of some imprint genes in the oocytes or embryos from IVM suggested that IVM procedure might influence the DNA methylation during the oocyte maturation in-vitro [12–14]. However, the influence of IVM on the process of histone modification in oocyte, another important mechanism in epigenetic reprogramming, has not been documented. 

Histone modification includes acetylation and methylation of lysines (K) and arginines (R), phosphorylation of serines (S) and threonines (T), ubiquitylation of lysines, as well as ribosylation. Recent studies have shown that these histone modifications play important roles in the regulation of gene expression in mitotic cells. Some modifications such as acetylation of lysine residues in histones H3 upregulate transcription while other modifications like methylation of H3K9 downregulate transcription [15, 16]. Among all the modifications, histone acetylation happens most frequently [17]. Histone acetylation is catalyzed by histone acetyltransferases (HATs) that transfer acetyl groups from acetyl coenzyme A (acetyl-CoA) onto the *ε*-amino groups of conserved lysine residues within the core histones. The levels of histone acetylation in chromatin are determined by the cooperations of HATs and histone deacetylases (HDACs) [18]. 

Histone acetyltransferase GCN5 (GCN5), a type-A HAT, catalyzes the acetylation of nucleosomes in nuclei or free histones in cytoplasm and acts as transcriptional coactivator in gene regulation. Histone deacetylase 1 (HDAC1) is one of Rpd3-like HDACs [19, 20], first identified as an IL-2 inducible gene. Overexpression of HDAC1 could cause aberrant morphologies and a partial blockage in the G2/M phases of the cell cycle [21]. GCN5 and HDAC1 are crucial for epigenetic reprogramming, regulation of gene expression, and cell proliferation during embryo development, but their exact roles and the underlying mechanisms during oocytes maturation in vitro remain unclear. Lysine residues of the amino terminal tail domain of histone H3 are common targets for histone acetylation which results in an allosteric change in the nucleosomal conformation and an increased accessibility to transcriptional factors by DNA.

The active roles of GCN5 and HDAC1 in epigenetic reprogramming and embryonic development prompted us to examine if IVM procedure may have an impact on these enzymes' expressions and functions. We determined the expression and distribution of GCN5, HDAC1, and Ac-H3 in metaphase II oocytes matured in vitro and in vivo. To observe the extended effects of IVM, the levels of these factors were followed in the embryos from IVM and control groups.

## 2. Materials and Methods

### 2.1. Experimental Animals

Animal care and procedures were carried out following Institutional Guidance of the Laboratory Animal of the Animal Care of Usage Committee (ACUC) of Zhejiang University, and the protocol was approved by the ACUC of Zhejiang University School of Medicine. Female ICR mice (6-7 weeks old) and male mice (8–12 weeks old) were housed in 12/12-hour light/dark cycle at 25 ± 0.5°C and 50–60% humidity. The mice were fed ad libitum with a standard pellet diet and water. The female mice are divided into two groups randomly, IVM group and control group.

### 2.2. Collection of Oocytes

For IVM group, female mice (6-7 weeks old) received 5 IU PMSG (pregnant mare serum gonadotropin; Gestyl, Organon, Oss, The Netherlands) 46–48 h before being sacrificed by cervical dislocation. The ovaries were excised, and antral follicles were punctured with 27 G needles in HEPES-buffered human tubal fluid medium (MHTF, Irvin Scientific, Santa Ana, CA, USA) supplemented with 10% Quinn's Advantage Serum Protein Substitute (SPS, SAGE/CooperSurgical Inc., Trumbull, CT, USA). The cumulus-enclosed oocytes at GV stage were selected. Oocytes were matured as previously described [22, 23]. Briefly, GV stage oocytes were cultured in human tubal fluid (HTF, Irvin Scientific, Santa Ana, CA, USA) medium containing 10% SPS supplemented with 0.1 IU/ml follicle stimulating hormone (FSH, Gonal F, Serono, Aubonne, Switzerland) and 0.5 IU/ml human chorionic gonadotropin (hCG, Pregnyl, Organon, Oss, The Netherlands) for 16–18 hours at 37°C in a humidified atmosphere of 5% CO_2_. Oocytes were observed under microscopy, and the disappearance of germ vesicle and the extrusion of the first polar body were used as the criteria of the maturation of oocytes. 

For the control group, mice were superovulated as described previously [24]. Briefly, mice received intraperitoneal injection of 7.5 IU hCG 46–48 hrs after the administration of 7.5 IU PMSG. Mice were sacrificed by cervical dislocation 12–14 hours after hCG injection, and the oviducts were excised. Cumulus masses were recovered from the dilated ampullae under a dissecting microscope. The collected cumulus masses were digested with hyaluronidase to remove granulosa cells. The naked oocytes were either placed in a drop of HTF for in vitro fertilization or washed three times with phosphate-buffered saline (PBS) for oocyte fixation or mRNA extraction.

### 2.3. IVF-ET

Sperm collected from caudal epididymides of ICR male mice were allowed to disperse in 10% SPS HTF and incubated at the conditions of 5% CO_2_, 37°C for 1 hr. For IVF, matured MII oocytes from either IVM group or the control group were inseminated with 4 × 10^5^ sperm/ml in drops of 30 *μ*l of 10% SPS HTF. About 4–6 hrs after insemination, oocytes were removed from fertilization medium to fresh 10% SPS HTF [22]. 

Intact oocytes were cultured in fresh 10% SPS HTF under oil at 37°C in 5% CO_2_ in air. Fertilization was judged if the oocyte showed extrusion of second polar body or the appearance of bi-pronucleus 9 hrs after insemination. Two-cell, four-cell, and eight-cell embryos were harvested for fixation or mRNA extraction after being washed three times in PBS.

Estrous ICR female mice were mated with vasectomized males (1: 1) on the same day as IVF for preparing the pseudopregnant female mice. Embryos at the 2-cell stage were transferred into the oviducts (maximum of 15 per oviduct) of 0.5 d.p.c. pseudopregnant ICR recipient female mice anesthetized with 2.5% Avertin i.p. Recipients were kept warm on a heating pad until fully recovered from anesthesia [25, 26].

### 2.4. Quantitative Real-Time RT-PCR

All the pools were done in triplicate and contained 60 oocytes or embryos from different developmental stages: MII oocytes, 2-cell embryos, and 8-cells embryos [27]. The total RNA was extracted from those pools using Absolutely RNA Microprep Kit (Stratagene, La Jolla, CA, USA) as described by the manufacturer. The entire RNA pellet was used for the RT. RT-PCR was carried out using the SYBR PrimeScript RT-PCR Kit (Takara, China). 4 *μ*l 5X PrimeScript Buffer PCR buffer, 1 *μ*l PrimeScriptTM RT Enzyme Mix I, 1 *μ*l Oligo dT Primer (50 *μ*M), and 1 *μ*l Random oligos (100 *μ*M) were added to the pool to obtain a total reaction mix volume of 20 *μ*l reaction system. The mixture was incubated at 37°C for 15 min, and the reaction was inactivated at 85°C for 5 sec. RT products were amplified by real-time PCR with SYBR-Green I (Takara, China) on ABI real-time PCR system (Applied Biosystems) according to the manufacturer's instructions. GAPDH was used as an internal reference gene. Real-time PCR was carried out in 20 *μ*l reactions containing 10 *μ*l SYBR Premix Ex Taq, 0.4 *μ*l PCR Forward Primer (10 *μ*M), 0.4 *μ*l PCR Reverse Primer (10 *μ*M), 0.4 *μ*l ROX Reference Dye, and 2 *μ*l cDNA sample. PCR was performed with one denaturation cycle at 95°C for 10 sec and 40 amplification cycles at 95°C for 5 sec and 60°C for 30 sec. Primer sequences for the genes are shown in Table 1. Data were analyzed by the comparative threshold cycle (CT) method and the standard formula [28].

### 2.5. Fluorescence Immunocytochemistry

Immunofluorescent staining was conducted as previously described [29]. Following fixation in 4% formaldehyde (Sigma, St. Louis, MO, USA) at 25°C for 30 min, oocytes and embryos were permeabilized with 1% Tween-20 in PBS containing 0.1% BSA at 4°C for 60 min. Nonspecific binding was blocked with heat-inactivated sheep serum in PBS (30% v/v) containing 2% BSA at 25°C for 30 min. Oocytes and embryos were incubated with antibodies against HDAC1 (rabbit, 1: 200; Upstate Biotechnology Inc., Lake Placid, NY, USA), GCN5 (goat, 1: 200; Santa Cruz Biotechnology, Inc., Santa Cruz, CA, USA), or Ac-H3 (rabbit, 1: 800; Cell Signaling Technology, Inc., Danvers, MA, USA) at 4°C overnight. The cells were washed three times with PBS, incubated in fluorescein isothiocyanate- (FITC- ) labeled secondary antibody (rabbit antigoat IgG, 1: 200, for GCN5; goat anti-rabbit IgG, 1: 200, for HDAC1 or Ac-H3; Zhongshan Golden Bridge Biotechnology, Co., Beijing, China) at 25°C for 30 min. The slides were washed, and a drop of DAPI (Vector Laboratories, Burlingame, CA) was added. The slides were then sealed with a coverslip. The fluorescent signals were detected with a Laser-Scanning Confocal Microscopy (Zeiss, LSM 510 META, Jena, Germany). Instrument settings were kept constant for each replicate. The fluorescence images were analyzed by using the program Image-J from the National Institutes of Health (http://rsb.info.nih.gov/ij/) (USA). Each developmental panel was repeated three times, and at least 20 oocytes or embryos were evaluated each time. In each experiment, samples without primary antibody were included as negative controls.

### 2.6. Statistical Analyses

Chi-square test was applied for the comparison of fertilization and cleavage and early development rates. Data of fluorescence immunocytochemistry and RT-PCR between IVM and control groups was compared by independent-test using SPSS 16.0 (Statistical Package for the Social Science, SPSS Inc., Chicago, USA), and *P* < .05 was considered statistically significant.

## 3. Results

### 3.1. IVM Decreased the Rates of Fertilization, Cleavage, and Developmental Competence

**Figure 1:**
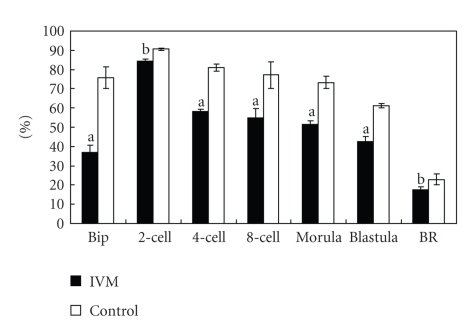
Rates of fertilization, cleavage, developmental competence, and birth. Bip: Bipronucleus; BR: total number of born mice/total number of 2-cell embryos for transplantation. (a) *P* < .01; (b) *P* < .05.

The rates of fertilization, cleavage, blastulation, and birth were significantly lower in IVM group than those in the control group (*P* < .05) (Figure 1).Meanwhile, 69.13% of 2-cell embryos in IVM group could develop to 4-cell embryos while 89.21% in the control group (*P* < .05).

### 3.2. The mRNA Expression of GCN5

**Figure 2:**
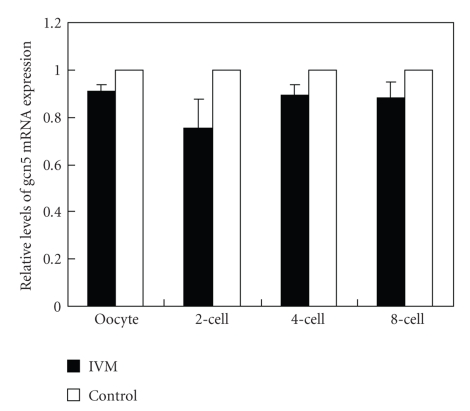
mRNA expressions of GCN5 in oocytes and embryos. The comparison of GCN5 mRNA expression was made between IVM and control groups. Summary data showed the relative expression levels of GCN5 in oocytes and embryos after real-time PCR analysis. The relative mRNA levels represent the amount of mRNA expression normalized with GAPDH.

The GCN5 mRNA was detected in both MII oocytes and embryos. The mRNA expressions were decreased in IVM oocytes; and embryos, however, no significant statistically differences were detected (*P* < .05) (Figure 2).

### 3.3. Localization and Intensity of GCN5

**Figure 3:**
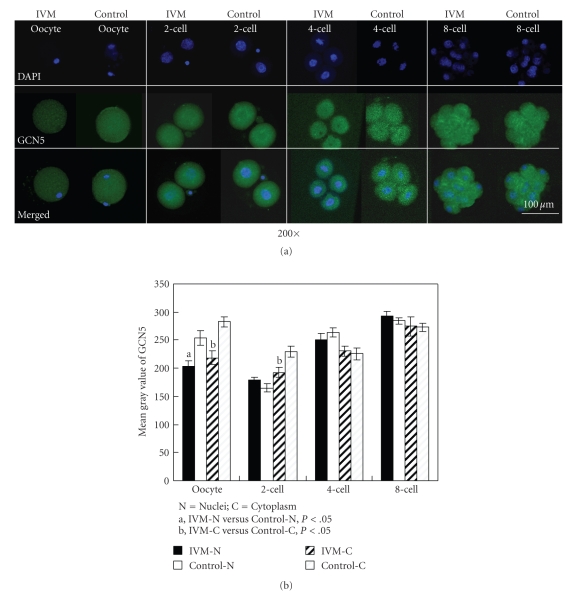
Fluorescence immunocytochemistry of GCN5 in oocytes and embryos. (a) Expressions of GCN5 in MII oocytes, 2-cell, 4-cell, and 8-cell embryos from IVM and control groups. Each sample was counterstained with DAPI (blue) to visualize the DNA. Specific goat polyclonal GCN5 was detected by fluorescein-conjugated antigoat secondary antibodies (green colour, FITC-labeled). Bar represents 100 *μ*m. (b) The gray value of GCN5 in oocytes and embryos.

Fluorescence immunocytochemistry showed that GCN5 was expressed in oocytes and embryos in both IVM and control groups. Quantitative analysis showed that the fluorescent signals in oocytes and the cytoplasm of blastomeres in 2-cell embryo in IVM group were significantly lower than those in the control group (*P* < .01). Meanwhile, in the 2-cell embryos of the control group, GCN5 expression was obviously around karyotheca while in the same stage of embryo from IVM, the distribution of the fluorescent staining of GCN5 was almost even. However, after 2-cell stage embryos, no significant difference was found in the fluorescent intensities and distributions of GCN5 (Figure 3). 

### 3.4. The mRNA Expression of HDAC1

**Figure 4:**
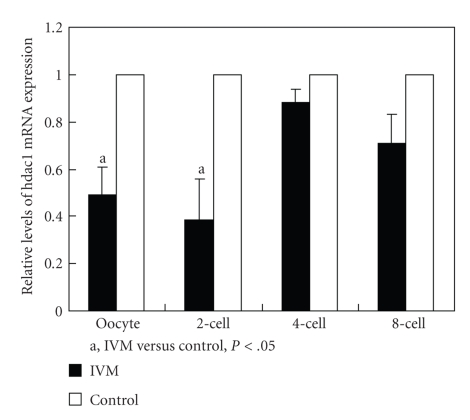
mRNA expression of HDAC1 in oocytes and embryos. The comparison of HDAC1 mRNA expression was made between IVM and control groups. Summary data showed the relative expression levels of HDAC1 in oocytes and embryos after real-time PCR analysis. The relative mRNA levels represent the amount of mRNA expression normalized with GAPDH.

The HDAC1 mRNA was expressed in both MII oocytes and embryos. The mRNA expression was significantly lower in the IVM group than that in the control group in oocytes and 2-cell embryos (*P* < .05) (Figure 4). This trend continues in the 4-cell and 8-cell stages, but the differences did not reach a statistically significant level.

### 3.5. Localization and Intensity of HDAC1

**Figure 5:**
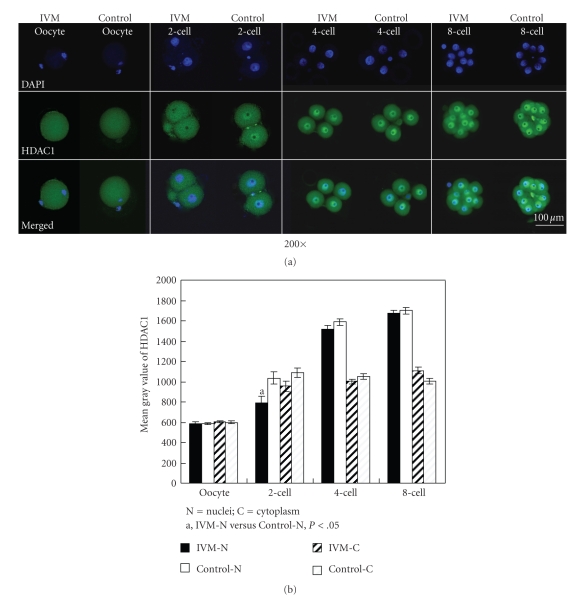
Fluorescence immunocytochemistry of HDAC1 in oocytes and embryos. (a) Expressions of HDAC1 in MII oocytes, 2-cell, 4-cell, and 8-cell embryos from IVM and control groups. Each sample was counterstained with DAPI (blue) to visualize the DNA. Specific rabbit polyclonal HDAC1 was detected by fluorescein-conjugated antirabbit secondary antibodies (green, FITC-labeled). Bar represents 100 *μ*m. (b) The gray value of GCN5 in oocytes and embryos.

The intensities and localizations of HDAC1 in the cytoplasm of MII oocytes and two-cell embryos were not significantly different between IVM and the control groups. But in the nuclei of 2-cell embryos, the mean gray value of HDAC1 in IVM group was significantly lower than that in the control group (*P* < .05). After 2-cell stage embryos, no significant differences were detected between IVM and the control groups either in the nuclei or in the cytoplasm (Figure 5).

### 3.6. Localization and Intensity of Ac-H3

**Figure 6:**
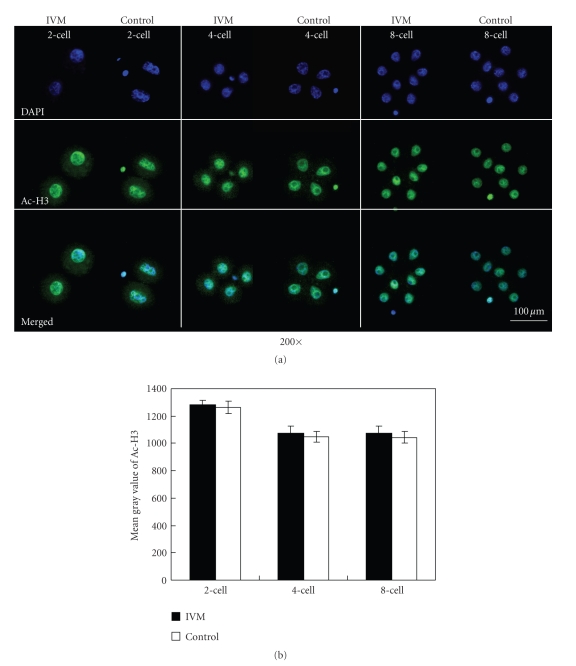
Fluorescence immunocytochemistry of Ac-H3 in oocytes and embryos. (a) Expression of Ac-H3 in 2-cell, 4-cell, and 8-cell embryos from IVM and control groups. Cells were immunostained with the Ac-H3 antibody. Each sample was counterstained with DAPI (blue) to visualize the DNA. Specific rabbit polyclonal Ac-H3 was detected by fluorescein-conjugated antirabbit secondary antibodies (green, FITC-labeled). Bar represents 100 *μ*m. (b) The gray value of GCN5 in oocytes and embryos.

No appreciable expression of Ac-H3 was observed in MII oocytes. Ac-H3 was only detected in the nuclei in embryos, and no significant differences of fluorescent intensities were found between IVM and the control groups (Figure 6).

## 4. Discussion

In this study, we found that histone acetylation in oocytes, early cleavage embryos, and (two-cell embryos) was changed in IVM group. The rates of fertilization, cleavage, and developmental competence from two-cell to four-cell stage were significantly lower in IVM group, so as the birth rate (BR) with two-cell transfer. We confirmed that IVM affected the quality of oocytes during the process of epigenetic reprogramming and led to the results above. 

Although the decreased expressions of GCN5 mRNA were detected in IVM group, no statistically differences were shown. The absence of change in GCN5 mRNA expression in both MII oocytes and two-cell embryos from IVM indicated that IVM might not affect GCN5 transcription, and the maternally inherited GCN5 mRNAs that were transcribed during the oocyte growth phase were abundant [30].

However, GCN5 protein expressions were decreased in IVM oocytes and two-cell embryos. These changes were most likely caused by the difference between a relatively simple in vitro culture condition and the complex in vivo system supported by multiple interactions of various factors and cells in ovary [31–33]. The polarity of blastomere was fundamental for the subsequent development and differentiation of the embryos [34–37]. It was reported that cytoplasm is polarized by cortical proteins, and this polarization then influences the stability of other maternally expressed proteins that in turn determine early embryonic cell fates [35]. In the present study, the distribution polarity of GCN5 protein in the blastomere of two-cell embryos disappeared in the IVM group, which might affect the subsequent development. The decreased expression and altered distribution of GCN5 in IVM oocytes and two-cell embryos suggested that IVM could disturb the function of GCN5 during the period of reprogramming, which might lead to epigenetic alterations. 

HDAC1 played a role in the ATP-dependent chromatin remodeling [38], participated in the downregulation of a variety of DNA-binding transcription factors [19, 39], and modulated cell proliferation. The significantly decreased HDAC1 mRNA levels in both oocytes and two-cell embryos from IVM strongly implied that IVM procedure downregulated the transcription of HDAC1 gene in oocytes before and after fertilization. During the growth of the mammalian follicular oocyte, the oocyte actively transcribed and produced stable RNA to support early embryonic cleavage. However, on the attainment of its full size, transcription ceased and the previously stored mRNA derived development through oocyte maturation, fertilization, and the early cleavage stages up to the activation of the embryonic genome [30]. In mice, the maternal/zygotic transition (MZT) occurs in the late two-cell stage. The defects of HDAC1 mRNA in MII oocytes suggested that the down-regulation of HDAC1 gene resulted from IVM occurred at the early stage of oocyte maturation. The low rate of developmental competence from two-cell to four-cell embryos could be explained by the delay of zygotic gene activation (ZGA) associated with expression alteration of HDAC1 and/or other genes [40].

Gioia et al. revealed that IVM oocytes failed to acquire full remodeling competence because of the disturbance of acetylation [41]. HDAC1 protein in cytoplasm of two-cell embryos was inherited from maternal storage while in the nuclei it was synthesized by the embryos themselves [42–45]. The reduced level of HDAC1 protein expression in nuclei of IVM 2-cell embryos suggested that IVM affected the HDAC1 protein syntheses and might interrupt its nucleus-cytoplasm distributions at this stage of embryos [20]. 

No differences were detected in the expression of GCN5 and HDAC1 after two-cell stage embryos, which indicated that the insufficiency induced by IVM might be rectified during the process of growing from two-cell to four-cell stage. The significantly lower rates of fertilization and cleavage in this IVM mouse model suggested that the mechanism of selection was functioned, and the oocytes and embryos seriously affected by IVM were eliminated before the embryo developed to two-cell stage. 

The level of acetyl-histone H3 is an index for the evaluation of global histone acetylation in chromatin. H3 acetylation is involved in gene expression regulation and genome reprogramming in oocytes and embryos [46, 47]. Although IVM could affect the expression and distribution of GCN5 and HDAC1 in MII oocytes and 2-cell embryos, which might be one reason for the low BR after 2-cell transfer, the levels of acetyl-histone H3 were not significantly changed in IVM group. Thus, global histone acetylation levels remain comparable in IVM oocytes and embryos. Other acetyltransferases or deacetylases may complement the function of GCN5 and HDAC1 on regulation of histone modification [48]. The global changes of acetylation in these residues of histone or specific changes in a target locus of chromatins associated with CGN5 and HDAC1 will require further investigation. The seeming inconsistency between GCN5/HDAC1 levels and H3 acetylation levels is partially caused by protein localization, as IVM-induced changes of GCN5 were observed in the cytoplasm, while Ac-H3 is associated with chromatins that localize in nuclei.

In conclusion, our studies showed that IVM could affect the expressions of GCN5 and HDAC1 in MII oocytes and two-cell embryos. However, these changes appear to be transient and normal levels resumed in later development stages. Although recent reports had shown that IVM might be safe to the newborn [49], the detrimental effects of IVM on the development of embryo, fetus, or even newborn could not be totally ruled out. Indeed, the lower pregnancy rate of IVM indicated that the impact of IVM procedures persist beyond the stage of implantation. The precise mechanisms by which IVM affect, the GCN5 and HDAC1 levels and the implication of these changes for the safety of offspring need to be investigated in future studies.

## Figures and Tables

**Table 1:** Reference genes selected for the study, and sizes of the PCR products.

	Genbank		

Gene name	Accession	Primer sequences	Product size (bp)
GCN5	NM_020004	5′-CGAGTTGTGCCGTAGCTGTGA-3′	96
		5′-ACCATTCCCAAGAGCCGGTTA-3′	
HDAC1	NM_008228	5′-CTGAATACAGCAAGCAGATGCAGAG-3'	92
		5′-TCCCGTGGACAACTGACAGAAC-3′	
GAPDH	NM_008084	5′–TGACGTGCCGCCTGGAGAAA-3′	98
		5′-AGTGTAGCCCAAGATGCCCTTCAG-3′	
